# Comparative Uptake and Distribution of Triclosan in Six Hydrophytes Grown in a Multi-Species Hydroponic System

**DOI:** 10.3390/plants15213477

**Published:** 2026-11-06

**Authors:** Yinli Shan, Nan Zheng, Yupeng He, Enguang Nie, Haiyan Wang

**Affiliations:** 1 School of Construction Art, Zhejiang College of Construction, Hangzhou 311231, China; 2 Key Laboratory of Nuclear Agricultural Sciences of Ministry of Agriculture, Institute of Nuclear Agricultural Sciences, Zhejiang University, Hangzhou 310058, China; 3 College of Bioscience and Biotechnology, Yangzhou University, Yangzhou 225009, China

**Keywords:** triclosan, phytoaccumulation, aquatic plants, interspecific competition, isotope tracing

## Abstract

Pharmaceutical and Personal Care Products (PPCPs) are emerging aquatic contaminants. Existing studies investigating the uptake of PPCPs by aquatic plants under hydroponic conditions have mostly been performed in non-competitive systems. Few reports are available regarding plant uptake and translocation of PPCPs under multi-species coexistence and interspecific competition. In this study, competitive hydroponic experiments were conducted in a greenhouse using ^14^C-triclosan to explore the uptake and distribution of triclosan in six hydrophytes: water spinach, purple perilla, cress, penny grass, cane shoot, and rice. The results showed that the triclosan uptake capacity of aquatic plants in the competitive hydroponic system followed the order: cress > water spinach > cane shoot > rice > penny grass > purple perilla. The root ^14^C-triclosan concentrations in water spinach (222.15–564.54 μg g^−1^) and cress (198.66–569.25 μg g^−1^) were significantly higher than those of the other species. Triclosan mainly accumulated in roots (90.36–99.04% of total plant uptake), with shoot levels 2–3 orders of magnitude below roots. Compared with non-competitive hydroponic conditions, interspecific competition exerted no significant effects on the uptake, distribution and translocation of triclosan in plants. This work advances the understanding of triclosan accumulation-translocation in hydrophytes under competitive conditions and supports risk assessment for triclosan.

## 1. Introduction

Pharmaceuticals and Personal Care Products (PPCPs) include many kinds of compounds that are widely used in the environment [1]. The potential adverse effects of these emerging types of pollutants on the environment, ecosystems, and humans have been reported in several studies [2,3,4]. Triclosan (TCS), an extensively used antimicrobial additive, has been widely found in rivers and lakes with concentrations ranging from 338 to 2300 ng L^−1^ [5,6,7] and from 3800 to 16,600 ng L^−1^ in influent wastewater [8,9], urine [10,11], blood plasma [10], and breast milk of pregnant women [12]. The widespread occurrence of TCS in aquatic environments has raised concerns regarding its potential ecological impacts. In the United States, TCS is regulated by the Environmental Protection Agency for certain pesticidal uses [13]. Similarly, the European Union has not approved TCS as an active substance for use in product-type 1 biocidal products intended for human hygiene [14]. These regulatory measures, together with the continued detection of TCS in aquatic environments, highlight the importance of understanding its environmental fate and potential ecological risks.

Furthermore, triclosan can be detected in various agricultural crops and aquatic plants under wastewater irrigation, such as carrot and *Typha latifolia* [15,16,17]. Therefore, a potential exposure pathway of triclosan to animals and humans is their consumption of triclosan-contaminated vegetables [18].

Aquatic plants have attracted considerable attention for the phytoremediation of contaminated water due to their potential to remove pollutants through various mechanisms [19]. The uptake and distribution of triclosan in plants irrigated with reclaimed water or cultivated in contaminated water have been documented in several studies [20,21]. For example, Macherius et al. found that the concentration of triclosan was 2.82 μg g^−1^ in the peels of carrot roots, but only 0.23 μg g^−1^ in carrot leaves after 60 days of growth in soil with 10 mg kg^−1^ triclosan [22]. Wu et al. showed that the concentration of triclosan in the roots of lettuce, spinach, cucumber and pepper ranged from 3.2 to 560 μg kg^−1^, which was much higher than its concentration in leaves (0–1.3 μg kg^−1^) after 21 days of growth in a nutrient solution with triclosan at 5 μg L^−1^ [23]. Mathews et al. reported that the translocation rate of triclosan from roots to shoots was less than 3.7% in cucumber, tomato, cabbage, okra, pepper, and potato after 30 days of growth in a nutrient solution containing 500 μg L^−1^ triclosan [24]. These reports demonstrated that triclosan accumulated mostly in plant roots rather than in other plant tissues. In addition to studies on terrestrial crops, aquatic plant-based treatment systems have been investigated for TCS removal. For example, Chand et al. reported differences in TCS removal efficiencies and associated rhizosphere microbial communities between Typha and Colocasia [25]. Moreover, the influence of interspecific competition on TCS accumulation and distribution among coexisting hydrophytes remains insufficiently understood [26,27].

Our previous research studied the uptake and distribution of triclosan in six kinds of aquatic plants (water spinach (*Ipomoea aquatica* Forssk.), purple perilla (*Perilla frutescens*), cress (*Oenanthe javanica* (Blume) DC.), penny grass (*Hydrocotyle vulgaris*), cane shoot (*Zizania latifolia* (Griseb) Stapf), and rice (*Oryza sativa*)) in a non-competitive hydroponic system, and found that the concentration of triclosan in the roots of water spinach (35–59 µg g^−1^) and cress (33–63 µg g^−1^) was remarkably higher than those in the other four plants, which followed the order of penny grass (7.7–60 µg g^−1^) > rice (2.9–28 µg g^−1^) > cane shoot (3.9–14 µg g^−1^) > purple perilla (2.8–9.4 µg g^−1^) [28]. The concentration of triclosan in the roots was 2–3 orders of magnitude greater than its concentration in the shoots of rice (0.1–0.5 µg g^−1^) > cane shoot (6.9 × 10^−2^–0.2 µg g^−1^) > cress (5.5 × 10^−2^–0.2 µg g^−1^) > water spinach (4.3 × 10^−2^–1.4 × 10^−1^ µg g^−1^) > penny grass (1.9 × 10^−2^–1.2 × 10^−1^ µg g^−1^) > purple perilla (1.1 × 10^−2^–4.0 × 10^−2^ µg g^−1^), and the range of triclosan content in the roots was 94.3–99.0% of its total accumulation in plants. However, previous studies have studied the uptake and accumulation of triclosan in plant systems; however, most of the studies have concentrated on a single plant species or non-competitive cultivation conditions. Few studies have investigated the behavior of triclosan in multispecies systems, and the influence of interspecific competition on the uptake, distribution, and translocation of triclosan among different hydrophytes remains unclear. Therefore, knowledge on the accumulation of triclosan under realistic multi-species coexistence conditions is important for the accurate evaluation of the environmental fate and ecological risk of triclosan.

In this study, we investigated the uptake and translocation of triclosan in six common hydrophytes in a competitive (multi-species co-cultured) hydroponic system using ^14^C-labeled triclosan. The concentration of ^14^C-triclosan in plant tissues was quantified by a liquid scintillation counter. In natural aquatic ecosystems, multiple plant species coexist and compete for resources, potentially influencing contaminant fate. By employing these six species as competitors, this experimental design enables assessment of interspecific competition effects on triclosan uptake. We hypothesize that, while competition may modulate triclosan bioavailability, intrinsic plant physiological characteristics remain the primary determinant of triclosan uptake and root-to-shoot translocation in the hydrophytes examined. This study provides critical data for refining environmental risk assessments of triclosan in aquatic systems.

## 2. Results

### 2.1. ^14^C-TCS Removal Rates from Nutrient Solution

**Figure 1:**
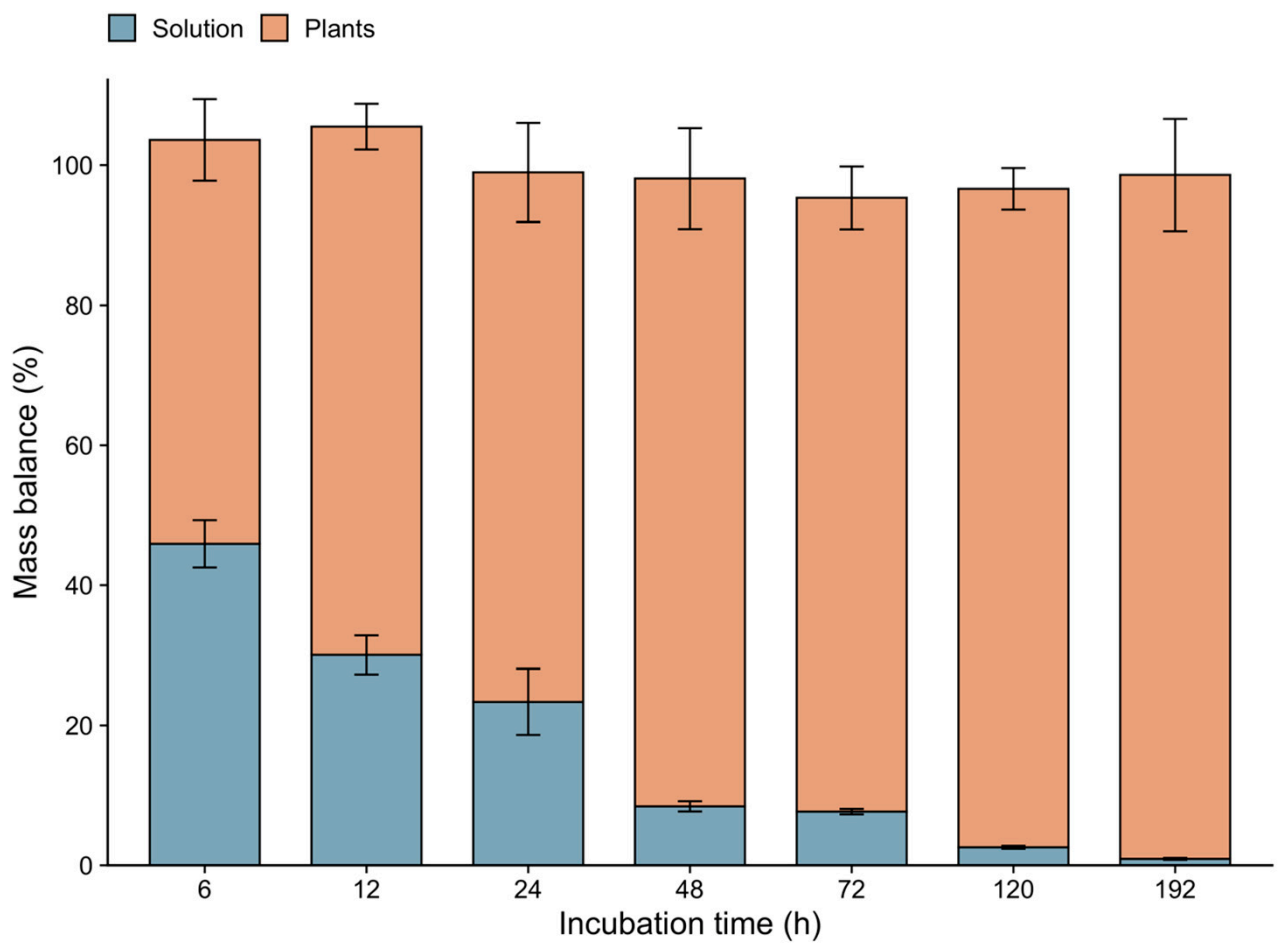
The mass balance of ^14^C-TCS in the competitive system.

Figure 1 shows the mass balance of ^14^C-triclosan in a competitive hydroponic system over the exposure time after triclosan treatment, and this mass balance was calculated using Equation (1) in Section 4.5. During the whole exposure of 192 h (8 days), the ^14^C-mass recoveries ranged from 95.3% to 103.6% of the initially applied radioactivity, indicating that almost all ^14^C-triclosan was taken up by the aquatic plants from the nutrient solution.

The amount of ^14^C-triclosan in plants was the summation of ^14^C-triclosan taken up by the six hydrophytes in one system; it increased notably over the exposure time and reached maximum levels at 192 h. The removal rate of ^14^C-triclosan was as high as 57.70% at 6 h, and increased significantly up to 72 h, then increased slightly to 97.68% at 192 h (Figure 1, *p* < 0.05), suggesting these six plants could be potentially used in phytoremediation of triclosan-contaminated water.

### 2.2. The BCF of ^14^C-TCS in Plants

**Figure 2:**
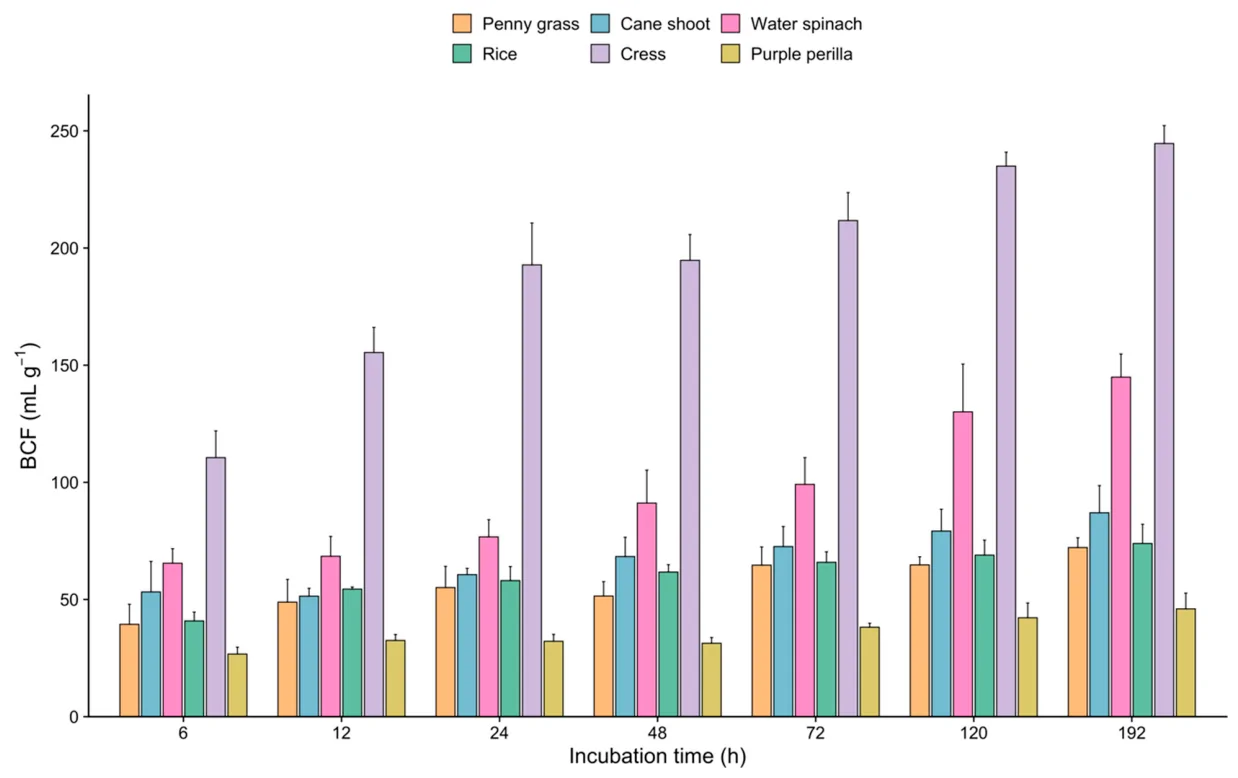
BCF of ^14^C-TCS in plants in a competitive hydroponic system.

Figure 2 shows that the BCF of ^14^C-triclosan in water spinach, purple perilla, cress, penny grass, cane shoot and rice were all significantly (*p* < 0.05) increased over incubation time. The BCF of ^14^C-triclosan in cress was 2–5-fold higher (*p* < 0.05) than that in the other five plants, which continually increased from 110.54 mL g^−1^ at 6 h to 244.60 mL g^−1^ at 192 h. The BCF in water spinach was lower than that in cress, but was notably (*p* < 0.05) higher than that in purple perilla, penny grass, cane shoot and rice, increasing remarkably (*p* < 0.05) from 65.48 mL g^−1^ (6 h) to 144.89 mL g^−1^ (192 h). This suggests that cress and water spinach have a higher bioconcentration capacity for TCS from water than the other six hydrophytes in the competitive hydroponic system. The BCF of ^14^C-triclosan in the other four plants followed the order of cane shoot (88.68 mL g^−1^) > rice (75.57 mL g^−1^) > penny grass (71.92 mL g^−1^) > purple perilla (57.86 mL g^−1^) at 192 h. The results reported here were consistent with the results we obtained in the non-competitive hydroponic system, which also demonstrated that the BCF of ^14^C-triclosan in cress (438.79 mL g^−1^ at 192 h) and water spinach (268.75 mL g^−1^ at 192 h) were remarkably (*p* < 0.05) higher than that in purple perilla, penny grass, cane shoot and rice, suggesting that the interaction between plants in a competitive system did not affect the uptake of ^14^C-triclosan in individual whole plant [28].

### 2.3. Distribution of ^14^C-Triclosan in Plants

The distribution of ^14^C-triclosan in plant shoots and roots was analyzed on a liquid scintillation counter, and the results were summarized in Table 1. Uptake and accumulation of ^14^C-triclosan in all plant roots and shoots increased over exposure time and reached their maximum levels at 192 h. The concentration of ^14^C-triclosan in the roots of water spinach and cress was significantly (*p* < 0.05) greater than that in the roots of the other four plants. For water spinach, the ^14^C-triclosan concentration in roots was 222.15 μg g^−1^ at 6 h, before slightly increasing to 336.22 μg g^−1^ at 72 h; it increased dramatically (*p* < 0.05) to 564.54 μg g^−1^ at 192 h. For cress, the ^14^C-triclosan concentration in roots increased notably (*p* < 0.05) from 198.66 μg g^−1^ (6 h) to 569.25 μg g^−1^ (192 h). The other four plants followed the order of penny grass (114.04–194.4 µg g^−1^) > purple perilla (59.89–117.20 µg g^−1^) > rice (51.91–93.34 µg g^−1^) > cane shoot (31.05–66.06 µg g^−1^). The results were consistent with the order of ^14^C-triclosan concentration in plant roots in a non-competitive system, in which water spinach (33–63 µg g^−1^) and cress (35–59 µg g^−1^) were also notably (*p* < 0.05) higher than penny grass (7.7–60 µg g^−1^) > rice (2.9–28 µg g^−1^) > purple perilla (2.8–9.4 µg g^−1^) > cane shoot (3.9–14 µg g^−1^).

The concentration of ^14^C-triclosan in rice shoots (2.22–7.82 µg g^−1^) was significantly (*p* < 0.05) greater than that in the other five plant shoots before 72 h, but the ^14^C-triclosan contents of 8.17–8.18 μg g^−1^ in the shoots of cane shoot were the highest at 120 h and 192 h. The ^14^C-triclosan concentration in shoots of water spinach (1.51–6.64 μg g^−1^) and cress (1.26–5.18 μg g^−1^) was higher than that in the shoots of penny grass (0.31–5.09 µg g^−1^) and purple perilla (0.15–1.64 µg g^−1^).

### 2.4. Implications to Human Exposure

The daily human exposure values of TCS ranged from 112.78 to 447.14 ng day^−1^ kg^−1^ (Table 2). The intake of TCS in water spinach (447.14 ng day^−1^ kg^−1^), penny grass (360.37 ng day^−1^ kg^−1^) and cress (330.72 ng day^−1^ kg^−1^) was significantly (*p* < 0.05) greater than purple perilla (112.78 ng day^−1^ kg^−1^).

## 3. Discussion

### 3.1. Species-Dependent ^14^C-Triclosan Uptake and the Limited Effect of Interspecific Competition

The observed ^14^C-triclosan removal pattern was consistent with our previous study of ^14^C-triclosan uptake by water spinach, purple perilla, cress, penny grass, cane shoot and rice in a non-competitive hydroponic system, which demonstrated that the ^14^C-mass recoveries ranged from 95.1% to 101.4% in the system, and cress and water spinach removed 94% and 96% of the ^14^C-triclosan from the system respectively at 192 h [28]. Liu et al. reported that more than 97% of triclosan could be removed from water by cattail, hornwort, and lesser duckweed (*Lemna minor*) [29]. Similarly, the average recoveries of ^14^C-triclosan were 90.3–99.6% in the entire incubation in a hydroponic celery system [30]. These results indicated that the formation of ^14^CO_2_ or volatile ^14^C-products from triclosan was minimal or absent in this hydroponic system.

The greater accumulation observed in water spinach and cress indicates species-specific differences in triclosan uptake capacity. The variation seen in the morphological structure of the roots, the surface area of the root, the growth pattern, and other physiological factors of the plants might affect the uptake of triclosan by the plants [31]. The similarity in species-specific accumulation patterns between the competitive and non-competitive systems suggests that interspecific interactions did not substantially affect the uptake and enrichment of ^14^C-triclosan in plant roots.

Although the uptake and accumulation patterns were similar to those previously observed under non-competitive conditions, the present study provides greater ecological relevance by examining different hydrophytes grown together under competitive conditions. Plants do not grow separately in their natural habitat; rather, they grow together and may be engaged in competition for nutrients, space, and contaminants. For this reason, the current study used the same six plant species but this time under competitive conditions [28]. As a result, the similarity of triclosan-accumulation trends in competitive and non-competitive conditions also implies that the physiological properties of plants were more significant for determining the amount of triclosan absorbed than interspecific competition. Thus, the uptake behavior of triclosan seems rather consistent under varying planting conditions.

### 3.2. Root Retention and Restricted Root-to-Shoot Translocation of Triclosan

The species-specific ranking of ^14^C-triclosan concentrations in plant shoots were also generally consistent with the order of ^14^C-triclosan concentration in plant shoots in a non-competitive system, rice (0.1–0.5 µg g^−1^) > cane shoot (6.9 × 10^−2^–0.2 µg g^−1^) > cress (5.5 × 10^−2^–0.2 µg g^−1^) > water spinach (4.3 × 10^−2^–1.4 × 10^−1^ µg g^−1^) > penny grass (1.9 × 10^−2^–1.2 × 10^−1^ µg g^−1^) > purple perilla (1.1 × 10^−2^–4.0 × 10^−2^ µg g^−1^) [28]. These results indicated that interspecific competition did not alter the root-shoot distribution or translocation of ^14^C-triclosan. Importantly, the competitive hydroponic system employed here offers ecological realism by mimicking multi-species interactions inherent to natural aquatic systems, which cannot be captured by single-species experimental designs.

The concentrations of ^14^C-TCS in plant roots in a competitive system were 2–3 orders of magnitude greater than those in shoots. For the total accumulation of ^14^C-triclosan in plants, the ^14^C-triclosan percentage in plant roots accounted for 90.36–99.04% (Table 3), and only a small quantity of ^14^C-triclosan transferred from roots to shoots. Persistence of ^14^C-triclosan within the roots suggests that triclosan accumulation predominantly occurred in the roots. This could be attributed to the hydrophobic nature of triclosan, which facilitates the entry of triclosan into lipophilic roots as well as its adsorption onto the roots. As such, only a small fraction of the absorbed triclosan was translocated to aerial parts, as has been demonstrated in other studies conducted in hydroponic plants [21,30]. It is beneficial for phytoremediation in that it increases contaminant removal in water while minimizing its movement to the aerial edible parts [32]. The results were consistent with our previous report in non-competitive hydroponic system, where the concentration of ^14^C-triclosan in the roots of water spinach (35–59 µg g^−1^), cress (33–63 µg g^−1^), penny grass (7.7–60 µg g^−1^), cane shoot (3.9–14 µg g^−1^), purple perilla (2.8–9.4 µg g^−1^) and rice (2.9–28 µg g^−1^) were also 2–3 orders of magnitude greater than their concentration in the shoots of rice (0.1–0.5 µg g^−1^), cane shoot (6.9 × 10^−2^–0.2 µg g^−1^), cress (5.5 × 10^−2^–0.2 µg g^−1^), water spinach (4.3 × 10^−2^–1.4 × 10^−1^ µg g^−1^), penny grass (1.9 × 10^−2^–1.2 × 10^−1^ µg g^−1^) and purple perilla (1.1 × 10^−2^–4.0 × 10^−2^ µg g^−1^), and the ^14^C-triclosan content in roots was 94.3–99.0% of the total accumulation in plants in a non-competitive system [28]. Our results in both competitive and non-competitive hydroponic systems were also consistent with the previous reports on xerophytes. For example, Macherius et al. documented that the concentration of triclosan was 2.82 μg g^−1^ in the peels of carrot roots, but only 0.23 μg g^−1^ in carrot leaves [22]. Wu et al. found that the content of triclosan in carrot root was more than 1 μg kg^−1^, but no triclosan was detected in the edible parts of celery, lettuce, spinach, cabbage, cucumber, bell pepper, and tomato after the plants were irrigated with treated wastewater under field conditions [16]. The limited translocation of triclosan from roots to shoots may be due to the high lipophilicity of triclosan [33,34,35,36] and the relatively higher lipid contents in roots [37]. The log K_ow_ of triclosan is 4.76 in neutral form [23]. Lipophilic compounds with log K_ow_ ≥ 5 can translocate very limitedly in plants due to the strong affinity with lipophilic domains in plants (lipids) [37,38,39]. In general, the amount of lipids in roots is more than that in other plant tissues [40], which makes roots act as the major reservoir for highly hydrophobic contaminants [41]. In addition, the limited translocation of organic contaminants within plants may also be due to the plant’s physiological structure, for example, the plant phloem. For long-distance movement, organic contaminants are mainly transported through the phloem within plants [42]. The phloem of penny grass and purple perilla is very narrow, which may contribute to the limited transportation of ^14^C-triclosan within the two plants [43,44]. The phloem in rice is relatively well-developed and may be helpful for the transportation of organic contaminants, including triclosan; this may contribute to the lower concentration of ^14^C-triclosan in the shoots of penny grass and purple perilla than the other four plants. Whereas the competitive hydroponic study yielded important insight into triclosan bioaccumulation in coexisting hydrophytes, the study was performed under laboratory-controlled conditions. However, the hydroponic system used in the current study is not able to reflect the natural aquatic ecosystem because of the presence of other variables such as microbial degradation, water flow rate, and other plant species. Thus, further studies need to be conducted in a field environment for the validation of the results. It is recommended that future research be undertaken in more challenging environments such as sediments-water dynamics as well as replicating plant communities to understand triclosan fate and potential for phytoremediation in natural aquatic ecosystems.

### 3.3. Implications for Selecting Hydrophytes for Triclosan Phytoremediation

Therefore, cress and water spinach can be used as phytoremediation materials for triclosan in both competitive and non-competitive hydroponic systems. The increased bioaccumulation in cress and water spinach could possibly be due to differences among the plant species in terms of their root structure, biomass generation, and physiological properties that affect the rate of contaminant absorption. Plants with well-developed root systems increase the surface area available for triclosan to attach and be absorbed, thus making bioaccumulation easier. This shows the significance of selecting the appropriate plants in enhancing phytoremediation processes to remove triclosan pollution from aquatic environments due to the fact that uptake and metabolism vary between plants [32].

### 3.4. Interpretation and Limitations of the Dietary Exposure Assessment

Contamination of ^14^C-TCS in the human body via consumption of the edible parts of contaminated plants poses a threat to human health because of the increased interest in triclosan and its metabolites due to environmental stability and possible toxicological effects [45]. The estimated daily human exposure was calculated by the same Equation (4) [42]. In this study, the EDI values of TCS ranged from 112.78 to 447.14 ng day^−1^ kg^−1^, while the exposure value was still much lower than the recommended acceptable daily intake (ADI), i.e., 83.0 μg kg^−1^ day^−1^ [42]. Therefore, vegetable consumption under the experimental conditions described in this study can be viewed as a possible human exposure route to triclosan, even though the calculated doses pose a minimal risk to human health. However, this study focused solely on triclosan’s dietary risks, neglecting potential risks from its metabolites. Therefore, future studies should prioritize investigating the combined dietary risks of TCS and its metabolites on human health.

## 4. Materials and Methods

### 4.1. Chemicals

**Figure 3:**
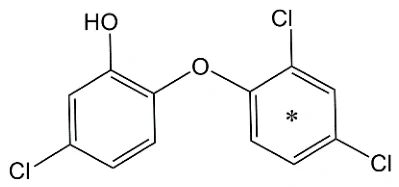
[Dichlorophenyl-U-^14^C]-labeled triclosan (^14^C atoms marked with *).

A supply of [dichlorophenyl-U-^14^C]-labeled triclosan (radiochemical and chemical purity > 99%, specific activity of 77 mCi mmol^−1^; see Figure 3 for structure and ^14^C labeling positions) was purchased from American Radiolabeled Chemicals (St. Louis, MO, USA). The radioactivity was quantified using a liquid scintillation counter (LSC, TriCarb-2910, PerkinElmer, Waltham, MA, USA) after mixing with 15 mL of scintillation cocktail I, which was prepared by dissolving 0.5 g of 1,4-bis-(5-phenyloxazol-2-yl)-benzene (POPOP, Arcos Organics, Geel, Belgium) and 5.0 g of 2, 5-diphenyloxazole (PPO, Arcos Organics) in 400 mL of 2-methoxyethanol (Sinopharm Chemical Reagent Co., Ltd., Shanghai, China) and 600 mL of dimethylbenzene (Sinopharm Chemical Reagent Co., Ltd., Shanghai, China).

### 4.2. Plants

The hydrophyte plants were water spinach (*Ipomoea aquatica* Forssk.), purple perilla (*Perilla frutescens* (L.) Britt.), cress (*Oenanthe javanica* (Blume) DC.), penny grass (*Hydrocotyle vulgaris* L.), cane shoot (*Zizania latifolia* (Griseb.) Stapf), and rice (*Oryza sativa* L.). The plants were cultured following the same method as described in our previous study: specifically, water spinach, cress and rice were germinated from seeds; cane shoot was germinated from old roots in vermiculite. Penny grass and purple perilla were purchased as intact plants from Fengqi Birdflower Market in Hangzhou, China. The plants were rinsed with distilled water and transplanted to a nutrient solution when the seedlings had grown for 3 to 4 weeks, and cultivated in a growth chamber at 20–25 °C (night-day), with a humidity of 80% and a day/night cycle of 12 h/12 h and constant aeration for a one-week acclimatization [28].

### 4.3. Multi-Species Hydroponic System

Six different types of plants were grown together in a single beaker of volume 100 mL, filled with 50 mL nutrient solution containing ^14^C-triclosan. For the purpose of this experiment, a single plant of each type was taken to represent interspecific competition. Such an experimental setup was chosen to study the effect of interactions between the plants on the uptake and distribution of triclosan. To reduce the photolysis of triclosan, all of the experimental beakers were wrapped with aluminum foil to minimize light penetration into the roots and nutrient solutions.

### 4.4. Plant Uptake and Sampling

Whole seedling samples were taken at the same time (6, 12, 24, 48, 72, 120, and 192 h after treatment) and treated in the same method as we used in our previous research [28]. Briefly, after sampling, the samples were washed with distilled water and separated into shoots and roots. Samples were oven-dried at 110 °C for 1 h to deactivate enzymes, then at 70 °C to constant weight. Dried samples were combusted in a biological oxidizer (OX-501 Biological Oxidizer, RJ Harvey Instrument Co., Tappan, NY, USA), and released ^14^CO_2_ was trapped in scintillation cocktail and quantified by a LSC.

### 4.5. Determination of ^14^C-Triclosan Concentration

The contents of ^14^C-triclosan in plant tissues were obtained according to the following Equation (1) [24].

(1) Concentration of C14−TCS= R⋅MV⋅C⋅S⋅1000

where *R* (dpm) is the ^14^C radiation value of nutrition solution measured by a liquid scintillation counter, *M* (mg mmol^−1^) is the molar mass of ^14^C-triclosan, *V* (mL) is the volume of nutrition solution, *C* is a convert unit 1mCi = 2.22 × 10^9^ dpm, and S (mCi mmol^−1^) is the specific activity of ^14^C-triclosan (77 mCi mmol^−1^).

### 4.6. Calculation of Mass Balance and Bioconcentration Factor

The mass balance of ^14^C-triclosan was calculated to evaluate the distribution of ^14^C-triclosan between plants and nutrient solution after exposure. The mass balance was calculated according to Equation (2)

(2) $$\mathrm{Mass}\begin{array}{c} \mathrm{blance}\begin{array}{c} \mathrm{of} \end{array} \end{array}C^{14}-\mathrm{TCS}(\%)=\frac{\;\begin{array}{c} P\begin{array}{c} +\begin{array}{c} N \end{array} \end{array} \end{array}}{A}\times 100\%$$

The bioconcentration factor (BCF) was calculated to evaluate the accumulation ability of different plant species according to Equation (3) [43]:

(3) BCF of C14-TCS (mL g−1)=CPCN

where C_P_ is the concentration of ^14^C-triclosan in plant tissues, and C_N_ is the initial concentration of ^14^C-triclosan in the nutrient solution.

### 4.7. Distribution Analysis of ^14^C-Triclosan in Plants

Based on Equation (1), we obtained the concentrations of triclosan in the roots and shoots of the samples, thereby determining its distribution across different plant tissues.

### 4.8. Human-Exposure Assessment

The human exposure assessment was carried out to determine the potential amount of exposure to triclosan-derived compounds by ingesting plant shoots containing this substance. The human exposure assessment (Estimated Daily Intake, EDI) was done to identify any dietary risk posed by the triclosan concentration in plant tissues [42,43].

(4) $$\mathrm{Human}\;\mathrm{exposure}\;(\mathrm{ng}{\mathrm{day}}^{-1}{\mathrm{kg}}^{-1})=\frac{C\cdot M\cdot V\cdot P}{D\cdot W}$$

In which C (nmol g^−1^) is the TCS concentration in fresh plant shoots in a competitive system, and the value used is the concentration measured at 192 h of exposure (shown in Table 2). M is the molar mass of TCS (289.5 g mol^−1^). V denotes the annual per capita consumption of total vegetables (94.1 kg) [46]. P is the percentage of leafy vegetables in total vegetables in China (33%) [47]. D is the number of days per year (365 days). W is the average weight of Chinese people, 61.75 kg [48].

### 4.9. Data Analysis

All the values were represented by mean ± SD of three replicate experiments, and significant values were taken when *p* < 0.05. Statistical analysis was performed using GraphPad Prism software (v10.0). The effects of different plant species on ^14^C-triclosan uptake and accumulation at each sampling time were analyzed by one-way analysis of variance (ANOVA). When there were significant differences among plant species (*p* < 0.05), Tukey’s HSD test was used for post hoc multiple comparisons.

## 5. Conclusions

This study investigated the uptake, distribution, and translocation characteristics of ^14^C-triclosan in various hydrophytes under a competitive hydroponic system. The results revealed that triclosan can be readily taken up by aquatic plants, and the uptake capacity for ^14^C-triclosan from water differed markedly among plant species, following the order cress > water spinach > cane shoot > rice > penny grass > purple perilla.

Under competitive hydroponic conditions, ^14^C-triclosan was predominantly accumulated in plant roots, accounting for 90.36–99.04% of the total amount, while only a small portion was translocated to above-ground shoots. This distribution pattern was consistent with the results obtained from the non-competitive hydroponic system, which could be attributed to the high lipophilicity of triclosan and the relatively high lipid content in plant root tissues. In addition, the BCFs and concentration rankings of ^14^C-triclosan in plant roots and shoots were largely comparable between the competitive system and the non-competitive hydroponic system reported in previous studies. This suggests that interspecific plant interactions under the competitive hydroponic system exerted no significant effects on the uptake, enrichment, distribution, and translocation of ^14^C-triclosan in the tested hydrophytes.

Human health risk assessment results indicated that consumption of these aquatic plants poses negligible human-health risks even under highly triclosan-contaminated conditions. Collectively, this study clarifies the differences in triclosan accumulation and translocation in hydrophytes under competitive versus non-competitive hydroponic conditions. It offers new insights into the phytoremediation mechanisms of triclosan-polluted water and provides a basis for the systematic evaluation of the environmental behavior and human-exposure risks of triclosan in aquatic ecosystems.

## Figures and Tables

**Table 1:** Concentrations of ^14^C-triclosan in plant tissues (μg g^−1^) after treatment.

Plant		^14^C-Triclosan in Plant Tissues (μg g^−1^)						
		6 h	12 h	24 h	48 h	72 h	120 h	192 h
Water spinach	shoots	1.51 ± 0.14 ^Bb^	1.88 ± 0.23 ^BCb^	1.98 ± 0.52 ^BCDb^	2.18 ± 0.45 ^CDb^	2.88 ± 0.30 ^CDb^	5.37 ± 0.43 ^BCa^	6.64 ± 1.34 ^Aa^
	roots	222.15 ± 27.02 ^Ac^	258.74 ± 16.66 ^Ac^	272.45 ± 30.77 ^Ac^	301.67 ± 51.75 ^Ac^	336.22 ± 34.45 ^Abc^	447.48 ± 80.06 ^Aab^	564.54 ± 50.54 ^Aa^
Penny grass	shoots	0.31 ± 0.10 ^Cc^	0.70 ± 0.19 ^CDbc^	0.92 ± 0.19 ^CDbc^	1.62 ± 0.49 ^Dbc^	2.32 ± 0.76 ^CDb^	4.21 ± 1.19 ^Ca^	5.09 ± 0.98 ^ABa^
	roots	114.04 ± 19.03 ^Bc^	122.32 ± 5.21 ^Cc^	139.40 ± 13.44 ^Bbc^	155.55 ± 17.08 ^Babc^	160.72 ± 4.53 ^Babc^	172.16 ± 11.82 ^Bab^	194.40 ± 20.91 ^Ba^
Purple perilla	shoots	0.15 ± 0.04 ^Cc^	0.43 ± 0.03 ^Dbc^	0.40 ± 0.14 ^Dabc^	0.79 ± 0.24 ^Dabc^	0.08 ± 0.20 ^Dab^	1.46 ± 0.51 ^Da^	1.64 ± 0.27 ^Ba^
	roots	59.89 ± 7.81 ^BCa^	79.33 ± 6.39 ^Da^	83.16 ± 9.22 ^Ca^	82.08 ± 9.07 ^Ca^	96.89 ± 6.33 ^Ca^	107.96 ± 14.67 ^Ba^	117.20 ± 35.61 ^BCa^
Cress	shoots	1.26 ± 0.37 ^Bc^	2.74 ± 0.46 ^Bbc^	3.70 ± 0.64 ^ABab^	4.04 ± 0.59 ^Bab^	4.37 ± 0.65 ^BCab^	5.18 ± 0.58 ^BCa^	5.18 ± 1.25 ^ABa^
	roots	198.66 ± 24.45 ^Ad^	203.77 ± 14.03 ^Bd^	283.61 ± 3.52 ^Acd^	314.27 ± 23.98 ^Ac^	327.15 ± 24.35 ^Ac^	456.12 ± 53.52 ^Ab^	569.25 ± 40.87 ^Aa^
Rice	shoots	2.22 ± 0.28 ^Ac^	5.23 ± 0.56 ^Ab^	5.37 ± 1.19 ^Aab^	5.54 ± 0.53 ^Aab^	6.98 ± 0.39 ^Aab^	7.46 ± 1.07 ^ABab^	7.82 ± 0.39 ^Aa^
	roots	51.91 ± 5.46 ^Cc^	64.46 ± 3.14 ^DEbc^	73.39 ± 7.24 ^Cab^	76.55 ± 6.61 ^Cab^	79.87 ± 6.79 ^Cab^	85.2 ± 5.71 ^Bab^	93.34 ± 11.45 ^Ca^
Cane shoot	shoots	1.62 ± 0.20 ^Bc^	2.62 ± 0.52 ^Bbc^	2.93 ± 0.69 ^BCbc^	3.41 ± 0.39 ^BCbc^	5.86 ± 1.05 ^ABab^	8.18 ± 0.73 ^Aa^	8.17 ± 2.48 ^Aa^
	roots	31.05 ± 3.22 ^Dcb^	39.50 ± 6.07 ^Eb^	58.56 ± 5.48 ^Ca^	58.92 ± 1.97 ^Ca^	58.90 ± 3.12 ^Ca^	60.55 ± 7.20 ^Ba^	66.06 ± 7.97 ^Ca^

Notes: ^A−E^: represent significant inter-species differences (*p* < 0.05) for either shoots or roots; ^a−c^: indicate significant differences across incubation time points for each individual plant.

**Table 2:** ^14^C concentration of fresh plant shoots in competitive system and human daily exposure of TCS.

	Water Spinach	Penny Grass	Purple Perilla	Cress
C (nmol g^−1^)	1.12 ± 0.23 ^A^	0.90 ± 0.17 ^A^	0.28 ± 0.05 ^B^	0.83 ± 0.20 ^A^
Daily exposure of TCS (ng kg^−1^ day^−1^)	447.14 ± 90.51 ^A^	360.37 ± 69.39 ^A^	112.78 ± 18.28 ^B^	330.72 ± 79.58 ^A^

Notes: ^A,B^: represent significant inter-species differences (*p* < 0.05).

**Table 3:** Distribution of ^14^C-triclosan in plant roots (%) after treatment.

	^14^C-Triclosan in Plant Roots (%)						
	6 h	12 h	24 h	48 h	72 h	120 h	192 h
Water spinach	95.53 ± 0.73	94.37 ± 1.62	92.66 ± 2.48	96.13 ± 1.28	94.85 ± 0.70	91.95 ± 1.01	94.83 ± 0.76
Penny grass	98.59 ± 0.37	96.97 ± 0.89	97.28 ± 0.28	93.86 ± 1.66	92.96 ± 2.77	91.37 ± 2.53	93.67 ± 0.98
Purple perilla	99.04 ± 0.23	97.61 ± 0.25	98.77 ± 0.27	94.78 ± 1.49	95.20 ± 0.82	94.52 ± 1.17	90.36 ± 4.13
Cress	97.03 ± 0.74	94.99 ± 1.09	95.59 ± 1.01	97.55 ± 0.44	98.47 ± 0.31	98.25 ± 0.23	96.98 ± 0.66
Rice	95.53 ± 0.73	94.37 ± 1.62	92.66 ± 2.48	96.13 ± 1.28	94.85 ± 0.70	91.95 ± 1.01	94.83 ± 0.76
Cane shoot	98.59 ± 0.37	96.97 ± 0.89	97.28 ± 0.28	93.86 ± 1.66	92.96 ± 2.77	91.37 ± 2.53	93.67 ± 0.98

## Data Availability

The data presented in this study are available on request from the corresponding author.
